# Neural traits characterize unconditional cooperators, conditional cooperators, and noncooperators in group‐based cooperation

**DOI:** 10.1002/hbm.24717

**Published:** 2019-07-16

**Authors:** Thomas Baumgartner, Franziska M. Dahinden, Lorena R. R. Gianotti, Daria Knoch

**Affiliations:** ^1^ Department of Social Psychology and Social Neuroscience, Institute of Psychology University of Bern Switzerland

**Keywords:** cooperation, neural trait, public goods game, resting electroencephalography

## Abstract

Contributing to and maintaining public goods are important for a functioning society. In reality, however, we see large variations in contribution behavior. While some individuals are not cooperative, others are highly so. Still others cooperate only to the extent they believe others will. Although these distinct behavioral types clearly have a divergent social impact, the sources of heterogeneity are poorly understood. We used source‐localized resting electroencephalography in combination with a model‐free clustering approach to participants' behavior in the Public Goods Game to explain heterogeneity. Findings revealed that compared to noncooperators, both conditional cooperators and unconditional cooperators are characterized by higher baseline activation in the right temporo‐parietal junction, an area involved in social cognition. Interestingly, conditional cooperators were further characterized by higher baseline activation in the left lateral prefrontal cortex, an area involved in behavioral control. Our findings suggest that conditional cooperators' better capacities for behavioral control enable them to control their propensity to cooperate and thus to minimize the risk of exploitation by noncooperators.

## 1 INTRODUCTION

In modern human society, many of the pressing issues such as depletion of natural resources, intergroup conflicts, and security of basic social systems require numerous individuals to contribute to, or maintain, public goods. Despite the pervasiveness of cooperation, there is substantial heterogeneity in people's propensity to cooperate (e.g., Declerck & Boone, 2018; Epstein, Peysakhovich, & Rand, 2016; Fehr & Schurtenberger, 2018; Fischbacher, Gächter, & Fehr, 2001; Kurzban & Houser, 2001; Nowak & Highfield, 2011). We know from daily life that some individuals are very cooperative and pay large personal costs to benefit the common good. They cooperate even though others might not cooperate as much (“unconditional cooperators”)—leaving them at risk of exploitation by noncooperative others. Other individuals solely aim to maximize their own welfare; thus, they free‐ride on the cooperation of others (“noncooperators”). Still other individuals might be inclined to cooperate but are willing to do so only to a certain extent. They cooperate as much as they assume everyone else cooperates; hence, their cooperation level is conditional on their belief about others' cooperation (“conditional cooperators”).

Previous attempts to explain heterogeneity in cooperative behaviors mainly focused on demographic or psychological variables, such as stable personality traits, but yielded rather variable results (e.g., Epstein et al., 2016; Kurzban & Houser, 2001; Volk, Thöni, & Ruigrok, 2011). The observed inconsistencies (e.g., findings that could not be replicated) might be due to the subjective nature of the employed self‐report measures (personality questionnaires), which are prone to various biases, such as demand characteristics and social desirability (e.g., the tendency of participants to answer questions in a manner that will be viewed favorably by others). Hence, the use of objective individual trait measures might be beneficial in understanding individual differences in human cooperative behavior.

One such ideal trait measure is the task‐independent neural baseline activation measured by resting electroencephalography (EEG). Resting EEG can be measured objectively and demonstrates both high temporal stability (Cannon et al., 2012; Dünki, Schmid, & Stassen, 2000; Williams et al., 2005) and high specificity (i.e., the extent to which a given EEG pattern uniquely belongs to a given person; Näpflin, Wildi, & Sarnthein, 2007). Studies investigating its temporal stability revealed test–retest reliabilities of up to 0.8 over a period of 5 years, while those exploring its specificity revealed recognition rates of up to 99%. Due to high intraindividual stability and specificity, resting EEG provides an ideal neural trait marker to investigate interindividual differences in cooperative behavior. Furthermore, prior literature linking psychological processes to neural functioning shows that neural traits allow inferences about the psychological processes that underlie individual differences in behavior (e.g., Gianotti et al., 2009; Gianotti, Nash, Baumgartner, Dahinden, & Knoch, 2018; Hahn et al., 2015; Knoch, Gianotti, Baumgartner, & Fehr, 2010; Schiller, Gianotti, Nash, & Knoch, 2014; Studer, Pedroni, & Rieskamp, 2013).

To measure individual differences in group‐based cooperative behavior, we employed a well‐established and widely used paradigm for measuring cooperation in groups, that is, the four‐person public goods game (PGG). The PGG mimics a social situation where a group of people simultaneously face a choice between acting cooperatively and contributing to a public good—which increases the whole group's payoff but comes at a personal cost—and acting uncooperatively and thereby increasing one's personal payoff only. Several studies have confirmed the external validity of this paradigm and showed that participants' behavior in a PGG significantly predicts their cooperative behavior in everyday life (e.g., Bluffstone, Dannenberg, Martinsson, Jha, & Bista, 2015; Carlsson, Johansson‐Stenman, & Nam, 2014; Fehr & Leibbrandt, 2011; Rustagi, Engel, & Kosfeld, 2010).

In order to identify the full set of behavioral types that constitute the repertoire of group‐based cooperative behaviors among the participants of our study (*N* = 137), we used a fully data‐driven and model‐free classification approach. Furthermore, because people's contribution behavior can be influenced by their beliefs about others' contribution behavior (e.g., Fischbacher & Gächter, 2010; Neugebauer, Perote, Schmidt, & Loos, 2009), we additionally elicited their belief about the average contribution of the other participants. This belief allowed us to further characterize the distinct behavioral types that emerged from the data‐driven classification procedure.

Previous evidence from task‐dependent studies using fMRI indicates that brain regions associated with social cognition (e.g., temporo‐parietal junction [TPJ], dorsomedial prefrontal cortex [DMPFC]), emotional empathy (e.g., insula), and behavioral control processes (e.g., lateral regions of the prefrontal cortex) play an important role in cooperation and prosocial behavior. For example, previous studies demonstrated an association between task‐dependent activation of the TPJ and generous choices in a donation task (Hare, Camerer, Knoepfle, O'Doherty, & Rangel, 2010), cooperative choices in a Prisoners' Dilemma Game (Rilling, Sanfey, Aronson, Nystrom, & Cohen, 2004), and altruistic choices in different versions of the Dictator Game (Hutcherson, Bushong, & Rangel, 2015; Park et al., 2017; Strombach et al., 2015). Furthermore, structural brain characteristics of the TPJ have been shown to be associated with altruistic choices in a Dictator Game (Morishima, Schunk, Bruhin, Ruff, & Fehr, 2012). Further, it has also been shown that task‐dependent activations in brain areas associated with affective sharing and empathy, such as the anterior insula, drive altruistic acts in empathy tasks (Hein, Silani, Preuschoff, Batson, & Singer, 2010; Lamm, Decety, & Singer, 2011; Tusche, Boeckler, Kanske, Trautwein, & Singer, 2016). Finally, various task‐dependent studies demonstrated that lateral areas of the prefrontal cortex are involved in strategic choices in fairness norm compliance (e.g, Tusche et al., 2016), and in the Prisoner's Dilemma Game (e.g., Fermin et al., 2016; Steinbeis, Bernhardt, & Singer, 2012). These task‐dependent studies are complemented by task‐independent studies demonstrating that structural brain characteristics of the lateral PFC are linked to strategic choices in a Dictator Game with and without punishment threat (Suzuki, Niki, Fujisaki, & Akiyama, 2011). Moreover, one recent patient study investigated group‐based cooperation using the PGG (Wills, FeldmanHall, NYU PROSPEC Collaboration, Meager, & Van Bavel, 2018). This study showed that patients with lesions in the lateral prefrontal cortex are less likely to cooperate.

However, although these previous studies help to understand the neural mechanism of cooperation or prosocial behavior, none of these studies used a cluster‐based approach to disentangle distinct behavioral types in a PGG. Accordingly, we know little about the distinct neural traits that allow the characterization of the behavioral types in group‐based cooperation. Since we applied a model‐free cluster approach we did not know a‐priori how many and what kind of behavioral types we would find. However, based on the findings mentioned above, we speculate that unconditionally cooperative types in the PGG might show high levels of baseline activation in the TPJ and/or DMPFC and conditional types might show more lateral PFC baseline activation due to the strategic nature of conditional group‐based cooperation. Since the previous studies only allowed for tentative hypotheses, we conducted whole‐brain corrected analyses to uncover the neural traits of different behavioral types in the PGG.

## 2 MATERIALS AND METHODS

### 2.1 Sample and procedure

We measured neural baseline activation and cooperative behavior in 137 healthy individuals (mean age = 21.1; *SD* = 3.0, 105 female). We recruited participants for one academic year in order to collect as many participants as possible during that time. Note that the EEG data collection and the behavioral data collection (PGG) were conducted in different sessions (see below for details). Data were analyzed after the collection was complete. Note that this study is part of a larger project (Gianotti, Dahinden, Baumgartner, & Knoch, 2019; Gianotti, Lobmaier, Calluso, Dahinden, & Knoch, 2017). Four participants (three female) were excluded from analysis because of technical problems during EEG or behavioral recordings. All participants were right‐handed and had no history of neurological or psychiatric disorders or alcohol and drug abuse. The study was approved by the local ethics committee and conducted according to the principles expressed in the Declaration of Helsinki. All participants gave written informed consent and were informed of their right to discontinue participation at any time. Participants received 40 Swiss francs (CHF 40; CHF 1 ≈ USD 1) for participating, in addition to the money earned in the cooperation paradigm. The EEG data collection and the measurement of group‐based cooperative behavior took place in different sessions and were separated by several weeks (mean = 13.8, *SD* = 8.7). When including the time lag between the two data collections as a covariate in the analyses, the results are not affected (see Table S1).

### 2.2 EEG recording and processing

For EEG data collection, participants were individually invited to the EEG laboratory. At the beginning of the session, participants gave written informed consent and completed a handedness inventory (Chapman & Chapman, 1987). Participants were then seated in a sound‐attenuated and electrically shielded chamber that was dimly lit and contained an intercom connection to the experimenters. EEG was recorded during rest with open or closed eyes; the instructions for eye‐opening/closing were given via intercom. The protocol consisted of 20 s with the eyes open followed by 40 s with the eyes closed, repeated five times (such a protocol guarantees minimal fluctuations in participants' vigilance state). In line with previous neural trait studies (Baumgartner, Gianotti, & Knoch, 2013; Gianotti et al., 2009; Kam, Bolbecker, O'Donnell, Hetrick, & Brenner, 2013; Li et al., 2017; Vecchio et al., 2013), data analysis were based on the 200‐s eyes‐closed condition.

EEG was recorded from 60 Ag/AgCl electrodes arranged in a 10–10 system montage (Nuwer et al., 1998) at a sampling rate of 500 Hz (bandwidth: 0.1–250 Hz). FCz and CPz were the recording and ground electrodes, respectively. Horizontal and vertical electro‐oculographic signals were recorded with electrodes at the left and right outer canthi and one electrode at the right infraorbital area. Eye‐movement artifacts were corrected using independent component analysis. EEG signals from channels with corrupted signals were interpolated. Computerized artifact rejection was applied (maximal voltage step: 15 μV; maximal amplitude: ± 100 μV; minimal allowed activity in intervals of 100‐ms length: 0.5 μV), and the data were also examined visually to eliminate residual artifacts (e.g., large movement‐related artifacts). All available artifact‐free 2‐s EEG epochs were extracted and recomputed against the average reverence. On average, there were 87.1 epochs (*SD* = 16.7) available per person. A fast Fourier transformation (using a square window) was applied to each epoch and channel to compute the power spectra with 0.5 Hz resolution. The spectra for each channel were averaged over all epochs for each participant. Absolute power values were integrated for the following seven independent frequency bands, according to Kubicki and colleagues (Kubicki, Hermann, Fichte, & Freund, 1979): delta (1.5–6 Hz), theta (6.5–8 Hz), alpha1 (8.5–10 Hz), alpha2 (10.5–12 Hz), beta1 (12.5–18 Hz), beta2 (18.5–21 Hz), and beta3 (21.5–30 Hz).

### 2.3 Intracortical source localization

The intracerebral electrical sources that generated the scalp‐recorded EEG activity were estimated with sLORETA (standardized low‐resolution electromagnetic tomography; Pascual‐Marqui, 2002). This method is a discrete, 3D distributed, linear, minimum‐norm inverse solution that computes electric neuronal activity as current density (A/m^2^) without assuming a predefined number of active sources. The sLORETA solution space consists of 6,239 voxels (voxel size: 5 × 5 × 5 mm^3^) and is restricted to cortical gray matter and the hippocampi, as defined by the digitized Montreal Neurological Institute (MNI) probability atlas. The sLORETA method has been validated in several studies combining EEG/MEG source localizations with other localization methods, such as functional Magnetic Resonance Imaging (e.g., Nuwer et al., 1998; Vecchio et al., 2013) and Positron Emission Tomography (e.g., Mobascher et al., 2009). Further, the method has been validated with experimental data for true generators, invasive implanted depth electrodes, whose locations were known (Zumsteg, Friedman, Wieser, & Wennberg, 2006; Zumsteg, Lozano, Wieser, & Wennberg, 2006). Using the automatic regularization method in the sLORETA software, we chose the transformation matrix with the signal‐to‐noise ratio set to 10. To reduce confounds without regional specificity, sLORETA images were normalized for each participant to a total power of one and then log‐transformed before statistical analyses.

### 2.4 The public goods game

Behavioral data collection took place at a behavioral laboratory with 24 interconnected computer terminals. Participants were randomly assigned to cubicles where they could take their decision in complete anonymity from the other participants. They were then randomly assigned to groups of four, endowed with 20 points (1 point = CHF 0.5) each, and faced with the decision (one‐shot) to either keep their endowment or contribute all or part of it to a public good (0–20 points). Each point contributed was doubled by the experimenter and the sum divided equally among all group members. Hence, each point contributed increased the aggregate group payoff but decreased the contributing individual's payoff. Note that doubling the contributions is the most classical and widely used multiplier in the PGG. Immediately after the contribution decision, participants reported their belief about the average contribution of the other three group members (0–20 points). The participants' final payoff in the PGG consisted of the earnings they gained from the public good and the points they had not contributed. Participants received written instructions and control questions ensured their understanding of the game.

### 2.5 Personality questionnaire

At the end of the behavioral session, participants completed the Honesty–Humility subscale of the HEXACO personality framework (Ashton & Lee, 2007), which has been associated in some studies with individual differences in cooperative behavior (e.g., Zumsteg, Lozano, et al., 2006). The Honesty–Humility subscale is a 10‐item questionnaire that measures participants' level of fairness, sincerity, greed avoidance, and modesty on a five‐point Likert scale.

### 2.6 Statistical analyses

First, we classified individuals into meaningful behavioral types based on their cooperative behavior. For this purpose, we applied the two‐step cluster analysis in SPSS (version 24.0). This clustering procedure divides participants into different clusters based on similarity/dissimilarity in their behavior. Importantly, this algorithm is thus naïve to the researchers' assumptions about the probable number of clusters/types as it automatically determines the optimal number of clusters. The optimal number of clusters (i.e., the minimal number that best accounts for the variability in the data) is automatically determined by a two‐step procedure. The first step calculates the Schwarz‐Bayesian information Criterion (BIC) for each number of clusters within a specified range and uses it to find the initial estimate for the number of clusters. The second step refines the initial estimate by finding the largest relative increase in distance between the two closest clusters in each clustering stage. The resulting statistics of the obtained cluster solution proved its good quality (silhouette measure of cohesion and separation = 0.7). Note that a silhouette measure above 0.5 corresponds to a good cluster solution.

Second, we conducted whole‐brain corrected analyses of variance (ANOVAs) in the different frequency bands to compare the neural baseline activations of the behavioral types that had emerged. Since ANOVAs are not implemented in the sLORETA software, normalized, and log‐transformed current density values for each voxel and participants were exported from sLORETA to Matlab. ANOVAs were then performed on each voxel using the anova1 function in Matlab and specifying the between‐subjects factor with three levels (three behavioral types). The corrections for multiple testing were incorporated using the nonparametric permutation tests described in Nichols and Holmes (Nichols & Holmes, 2001). In details, 5,000 permutations were run in order to estimate the empirical probability distributions. The statistical F‐images were then thresholded at the corresponding critical probability threshold (corrected for multiple comparisons at *p* < .05), and voxels with statistical values exceeding this threshold have their null hypotheses rejected. In order to control for potential gender effects, we performed all ANOVAs with gender as covariate. However, please note that our findings hold if we do not control for gender (see Table S2). Thus, gender did not affect our results.

Third, for regions that displayed significant, whole‐brain corrected differences between the three behavioral types, the respective voxel with the strongest effect was used as the center for spherical regions of interest (ROIs; radius: 10 mm). Averaged current density values were extracted for all voxels within these ROIs for visualization and further analyses (please see results section for details).

## 3 RESULTS

### 3.1 Emergence of the behavioral types

In order to identify the behavioral types present in our study sample, we conducted a two‐step cluster analysis of participants' cooperative behavior. This analysis yielded a solution with three distinctive clusters. The first cluster included 26 participants (19.5%) who demonstrated a very high level of cooperation; they contributed on average 19.0 points (95.0% of their endowment; *SD* = 1.8 points). The second cluster included 32 participants (24.1%) who demonstrated a very low level of cooperation; they contributed on average only 2.3 points (11.6% of their endowment; *SD* = 2.1 points). The third cluster included 75 participants (56.4%) who demonstrated a moderate level of cooperation; they contributed on average 9.4 points (47.1% of their endowment; *SD* = 2.2 points). Please note that we also ran an additional two‐step cluster analysis on participants' contribution and belief scores. Both cluster solutions led to the same optimal number of behavioral types and almost identical allocation of the participants to the three behavioral types (see Table S3 for details).

### 3.2 Characterization of the behavioral types

To characterize the behavioral types that emerged from the cluster analysis, we explored their cooperative behavior in relation to their beliefs about their counterparts' average contributions. This information helps to clarify the motives behind the cooperative or uncooperative behaviors of the distinct behavioral types. For example, did the individuals of the highly cooperative behavioral type contribute almost all of their endowment because they believed everyone would contribute a similar amount? Or did they contribute almost all of their endowment even though they suspected that the others would not contribute as much, thus being aware that their high level of cooperation could be exploited by others who would not be as cooperative? In order to clarify this, we generated a difference score by subtracting the value of participants' beliefs from their own contributions (contribution‐minus‐belief score).

**Figure 1:**
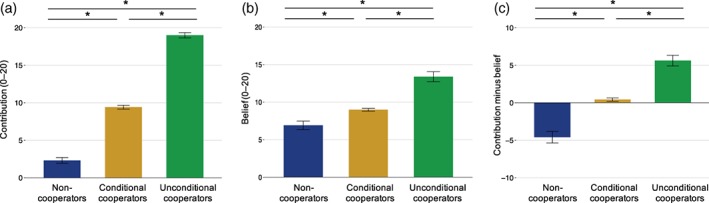
Contribution, belief and contribution‐minus‐belief scores of the three behavioral types. The bar graph in (a) illustrates the contribution level (0–20 points) of the three behavioral types that emerged from the model‐free cluster analysis. Unconditional cooperators (green) contributed on average 19.0 points to the public good, noncooperators (blue) contributed on average 2.3 points to the public good, and conditional cooperators (orange) contributed on average 9.4 points to the public good. The bar graph in (b) illustrates the level of participants' beliefs about the others' average contributions (0–20 points). Unconditional cooperators believed that the others contributed on average 13.4 points, conditional cooperators believed that the others contributed on average 9.0 points, and noncooperators believed that the others contributed on average 6.9 points. The bar graph in (c) illustrates the difference between participants' contribution and their beliefs about the others' contributions (contribution‐minus‐belief) by behavioral type. Unconditional cooperators contributed substantially more to the public good than they believed the others would contribute (positive value), noncooperators contributed substantially less to the public good than they believed the others would contribute (negative value), and conditional cooperators contributed as much to the public good as they believed the others would contribute (value close to zero). Error bars represent standard errors of the mean. The asterisks denote means that are significantly different from each other (at *p* < .05). Note that Figure S1 depicts these behavioral findings as box plots [Color figure can be viewed at http://wileyonlinelibrary.com]

The cluster that consisted of the participants with the highest level of contribution (*N* = 26) was characterized by positive values on the contribution‐minus‐belief score (mean = 5.6; *SD* = 3.7, see Figure 1) and these positive values were significantly different from zero (*t*‐test against zero: *t*[25] = 7.832, *p* < .001), indicating that these individuals contributed more to the public good than they believed the others would contribute. Furthermore, their contribution was not significantly correlated with their belief about the others' average contribution (*r* = .124, *p* = .545). Thus, it seems that these individuals did not condition their contributions on their beliefs about the others' contributions but instead even took the risk of being exploited by others. Hence, we refer to the individuals of this behavioral type as “unconditional cooperators.”.

In sharp contrast, the group of participants with the lowest level of contribution (*N* = 32) was characterized by negative values on the contribution‐minus‐belief score (mean = −4.6; *SD* = 4.3, see Figure 1) and these negative values were significantly different from zero (*t*‐test against zero: *t*[31] = −6.030, *p* < .001), indicating that these individuals contributed less to the public good than they believed the others would contribute. Furthermore, their contribution was not significantly correlated with their belief about the others' average contribution (*r* = −.247, *p* = .174). We refer to the individuals of this behavioral type as “noncooperators.”

Finally, the group of participants with moderate levels of contribution (*N* = 75) was characterized by values close to zero on the contribution‐minus‐belief score (mean = 0.4; *SD* = 1.9; *t*‐test against zero: *t*[74] = 1.948, *p* = .060, see Figure 1), indicating that these individuals contributed about as much to the public good as they believed the others would contribute. Indeed, their contribution was significantly correlated with their belief about the others' average contributions (*r* = .527, *p* < .001). Thus, it seems that the contribution level of these individuals depended on their beliefs about the others' contribution levels. Hence, we refer to the individuals of this behavioral type as “conditional cooperators.”

So far, we have characterized each of the three behavioral types separately, but we have not yet directly tested whether the behavioral types statistically differ in contributions, beliefs, and contribution‐minus‐belief scores. Thus, we next tested for differences between the three behavioral types. ANOVAs demonstrated a significant main effect for contributions (F[2,130] = 453.631, *p* < .001; explained variance = 87.5%; observed power = 100%; Figure 1a), beliefs (F[2,130] = 50.728, *p* < .001; explained variance = 43.8%; observed power = 100%; Figure 1b), and also for contribution‐minus‐belief scores (F[2,130] = 82.979; *p* < .001; explained variance = 56.1%; observed power = 100%; Figure 1c). Post hoc pairwise comparisons revealed significant differences between all three behavioral types in contributions (pairwise comparisons: all F > 240.840; all *p* < .001), beliefs (pairwise comparisons: all F > 20.077; all *p* < .001) and contribution‐minus‐belief scores (pairwise comparisons: all F > 70.490; all *p* < 0.001).

The three behavioral types did not differ in the personality traits of honesty and humility (ANOVAs: all *F*[2,129] ≤ 0.859, *p ≥* .426), nor in age (*F*[2,129] = 0.765, *p* = .468).

### 3.3 Neural trait signatures of the behavioral types

To examine whether the emerged behavioral types can be characterized by distinctive neural signatures, we conducted whole‐brain corrected source localization analyses that compared the task‐independent neural baseline activations between the behavioral types. For that purpose, a fast Fourier transformation was applied to compute the power spectra of each EEG channel. The resulting power values were integrated for the following seven independent frequency bands (Kubicki et al., 1979): delta (1.5–6 Hz), theta (6.5–8 Hz), alpha1 (8.5–10 Hz), alpha2 (10.5–12 Hz), beta1 (12.5–18 Hz), beta2 (18.5–21 Hz), and beta3 (21.5–30 Hz). The intracerebral electrical sources that generated the scalp‐recorded EEG activity were estimated for each frequency band with sLORETA (Pascual‐Marqui, 2002).This established method computes electric neural activity as current density in cortical gray matter (see methods section for details).

**Figure 2:**
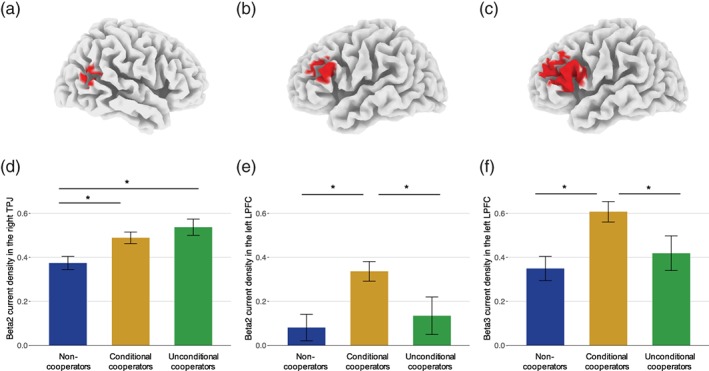
Brain areas of the right TPJ and lateral PFC demonstrate significant differences in baseline current density (a/m^2^) between the three behavioral types. Locations of the voxels in the right TPJ (a) and LPFC (b, c) that showed significant group differences (whole‐brain corrected at *p* < .05) in the beta2 and beta3 frequency bands are indicated in red. Bar graphs (based on ROIs encompassing all the voxels that showed significant differences) illustrate the baseline beta2 current density in the right TPJ (d), beta2 current density in the left LPFC (e), and beta3 current density in the left LPFC (f) in the three behavioral types. For display purposes, the current density data (log‐transformed) were converted to a positive scale (adding one to each value). Error bars represent standard errors of the mean. The asterisks denote means that are significantly different from each other (at *p* < .05). Note that Figure S2 depicts these neural findings as box plots. TPJ, temporo‐parietal junction TPJ; PFC, prefrontal cortex; LPFC, lateral prefrontal cortex [Color figure can be viewed at http://wileyonlinelibrary.com]

Analyses of variance (ANOVAs) revealed two brain areas that showed significant differences (whole‐brain corrected) between the behavioral types, the right temporo‐parietal junction in the beta2 frequency band (TPJ; cluster size: 15 voxels; BA: 22/39/40; peak MNI coordinates: *x* = 60, *y* = −60, *z* = 15; *F*[2,129] = 5.490, *p* = .005; explained variance = 7.8%; observed power = 84.3%; Figure 2a), and the left lateral prefrontal cortex in the beta2 and beta3 frequency bands (LPFC; beta2: cluster size: 19 voxels; BA: 9/45/46; peak MNI coordinates: *x* = −55, *y* = 25, *z* = 25; *F*(2,129) = 6.066, *p* = .003; explained variance = 8.6%; observed power = 87.9%; Figure 2b; beta3: cluster size: 72 voxels; BA: 9/10/45/46/47; peak MNI coordinates: *x* = −55, *y* = 25, *z* = 25; *F*(2,129) = 6.113, *p* = .003; explained variance = 8.7%; observed power = 88.2%; Figure 2c). There were neither significant differences between the distinct behavioral types in any other brain regions, nor in any other frequency band (all *p* > .268).

Pairwise comparisons between the behavioral types revealed a distinctive pattern in the right TPJ and left lateral PFC. As shown in Figure 2d, unconditional and conditional cooperators both showed higher beta2 current density in the right TPJ than noncooperators (unconditional cooperators vs. noncooperators: *F*(1,55) = 11.896, *p* = .001; explained variance = 17.8%; observed power = 92.3%; conditional cooperators vs. noncooperators: *F*(1,104) = 8.149, *p* = .005; explained variance = 7.3%; observed power = 80.7%). Interestingly, the two cooperative types did not differ with respect to beta2 current density in the right TPJ (*F*[1,98] = 0.527, *p* = .469).

As shown in Figure 2e,f, conditional cooperators showed higher beta2 and beta3 current density in the left lateral PFC than both noncooperators (beta2: *F*(1,104) = 9.886, *p* = .002; explained variance = 8.7%; observed power = 87.6%; beta3: *F*(1,104) = 10.125, *p* = .002; explained variance = 8.9%; observed power = 88.3%) and unconditional cooperators (beta2: *F*(1,98) = 5.613, *p* = .020; explained variance = 5.4%; observed power = 65.0%; beta3: *F*(1,98) = 4.961, *p* = .028; explained variance = 4.8%; observed power = 59.7%). Noncooperators and unconditional cooperators did not differ significantly with respect to beta2 or beta3 current density in the left lateral PFC (beta2: *F*(1,55) = 0.286, *p* = .595; beta3: *F*(1,55) = 0.557, *p* = .459).

Since the resting fast‐wave oscillations in the beta2 and beta3 bands likely reflect increased cortical activations (Gamma et al., 2004; Laufs et al., 2003; Oakes et al., 2004), these results indicate that both cooperative types (unconditional cooperators and conditional cooperators) were characterized by higher baseline activation in the right TPJ compared to noncooperators, while only conditional cooperators were also characterized by higher baseline activation in the left LPFC compared to either unconditional cooperators or noncooperators.

## 4 DISCUSSION

Human cooperative behavior is characterized by remarkable individual differences (e.g., Fischbacher et al., 2001; Nowak & Highfield, 2011). In the current study, cluster analysis identified three distinct behavioral types strongly differing in their contribution behaviors and beliefs about others' behavior in a PGG. Similar types have been reported in behavioral studies, using different classification approaches (model‐driven or model free; e.g., Epstein et al., 2016; Fallucchi, Luccasen, & Turocy, 2018; Fischbacher & Gächter, 2010; Fischbacher, Gächter, & Quercia, 2012; Frey, 2017; Gächter, Kölle, & Quercia, 2017; Kurzban & Houser, 2001, 2005). Furthermore, the frequencies of the behavioral types observed in our study also correspond nicely with the frequencies reported in the previous literature (see for example a recent re‐analysis of six large datasets by Fallucchi et al., 2018). By using a neural trait approach, we were able to characterize these three behavioral types by their neural signatures and shed light on possible underlying psychological mechanisms. The results showed that both cooperative types, unconditional cooperators, and conditional cooperators, were characterized by higher neural baseline activation in the right TPJ compared to noncooperators. However, only conditional cooperators were also characterized by higher neural baseline activation in the left LPFC compared to either noncooperators or unconditional cooperators.

Previous studies have consistently associated task‐dependent TPJ activation with aspects of social cognition, such as perspective‐taking, cognitive empathy, and self‐other distinction (Decety & Lamm, 2007; Frith & Frith, 2012; Jackson, Brunet, Meltzoff, & Decety, 2006; Lamm, Rütgen, & Wagner, 2017; Steinbeis, 2016; Tusche et al., 2016). Furthermore, task‐dependent activation of the TPJ has been associated with altruistic and generous choices (Hare et al., 2010; Hutcherson et al., 2015; Park et al., 2017; Strombach et al., 2015; Zanon, Novembre, Zangrando, Chittaro, & Silani, 2014). There has also been one previous structural MRI study (Morishima et al., 2012) that demonstrated a link between the gray matter volume of the TPJ and altruistic choices. Our finding that conditional and unconditional cooperators are characterized by increased baseline activation in the TPJ (compared to noncooperators) nicely complements this previous research and provides evidence that task‐independent baseline activation is associated with cooperative behavior in the PGG. We speculate that higher task‐independent baseline activation in the right TPJ is indicative of an individual's propensity to cooperate, possibly due to an increased capacity for social cognition processes that help to overcome one's self‐centered perspective.

Notably, the statistically indistinguishable baseline activation in the right TPJ of conditional and unconditional cooperators suggests that both behavioral types are characterized by a similar capacity for social cognition and propensity to cooperate. However, unconditional cooperators and conditional cooperators differed markedly in cooperative behavior and beliefs about others' cooperation. While conditional cooperators showed a restricted level of cooperation that was conditioned on their belief about others' cooperative behavior, unconditional cooperators contributed not only considerably more than conditional cooperators did, but also more than they believed the others would. In other words, they risked being exploited by less cooperative others. In contrast, conditional cooperators contributed only to the extent they believed others would contribute—thereby minimizing the risk of exploitation by noncooperators. Interestingly, these substantial differences in behavior and belief between the two cooperative types were paralleled by a neural trait difference: conditional cooperators were characterized by higher baseline activation in the left LPFC than unconditional cooperators. A large body of evidence has consistently linked the LPFC to behavioral control and self‐control processes (e.g., MacDonald, Cohen, Stenger, & Carter, 2000; Miller & Cohen, 2001), both in social and nonsocial contexts. Evidence from neuroimaging studies using task‐independent measures indicates that improved functioning of the LPFC is associated with enhanced capacities for behavioral control and self‐control processes (Crone & Steinbeis, 2017; Knoch et al., 2010; Schiller et al., 2014; Steinbeis et al., 2012; Yamagishi et al., 2016). Based on this research, we speculate that higher task‐independent baseline activation in the LPFC in conditional cooperators is indicative of their increased capacity for behavioral control and self‐control. We speculate that this control capacity might be critical in enabling conditional cooperators to adjust their high propensity to cooperate to a “reasonable” level, in the sense of a reduced risk of exploitation by others.

Taken together, our findings point to two capacities that seem to play a decisive role in determining the type of cooperative behavior. On the one hand, the capacity for social cognition seems to be fundamental to people's propensity to cooperate. On the other hand, as an excessively cooperative type is vulnerable to exploitation, additional capacity for behavioral control and self‐control might be essential to limit this propensity and thereby protect the individual from exploitation by noncooperators.

Although neural traits are stable, they are not unchangeable. Recent studies have documented that social cognition and behavioral control/self‐control capacities can be improved by specific behavioral trainings (e.g., meditation, repeated practices of working memory) or neuro‐modulation techniques (e.g., neurofeedback, tDCS) (Anguera et al., 2013; Houben, Dassen, & Jansen, 2016; Jaušovec & Jaušovec, 2012; Kouijzer, de Moor, Gerrits, Congedo, & van Schie, 2009; Santiesteban, Banissy, Catmur, & Bird, 2012; Valk et al., 2017). Moreover, training and neuro‐modulation induced changes in brain structure and function have been observed in regions of the lateral prefrontal cortex as well as the temporo‐parietal junction. Thus, it is conceivable that behavioral training and neuro‐modulation techniques that impact the brain regions involved in processes of social cognition and behavioral control/self‐control could help to promote cooperative behavior in noncooperative individuals and increase the number of individuals demonstrating a level of cooperation that is both reasonable for the individual and beneficial to society.

## AUTHOR CONTRIBUTIONS

T.B., L.G., and D.K. conceived and designed the study. T.B., L.G., and F.D. performed research. T.B. and F.D. analyzed the data. F.D., T.B., and D.K. wrote and L.G. commented the manuscript.

## CONFLICT OF INTERESTS

The authors declare that they have no competing interests.

## Supporting information


**Table S1 ANOVAs with and without covariate time lag.** Overview of the results of the ANOVAs testing the effect of behavioral types on neural baseline activation in the right TPJ (beta2), and the left LPFC (beta2 and beta3), calculated with and without including the time lag between the EEG and the behavioral data collection as a covariate.
**Table S2. ANOVAs with and without covariate gender.** Overview of the results of the ANOVAs testing the effect of behavioral types on neural baseline activation in the right TPJ (beta2), and the left LPFC (beta2 and beta3), calculated with and without including gender as a covariate.
**Table S3: Cluster solutions.** Overview of the allocation and the behavioral characteristics of the two cluster solutions.
**Figure S1: Box plots of the contribution (A), belief (B) and contribution‐minus‐belief score (C) of the three behavioral types.** This figure complements Figure 1 with additional statistical information. The box plot's lower and upper hinges correspond to the first and third quartiles (the 25th and 75th percentiles), the median is indicated by the thick horizontal line within the box plot. The whiskers extend from the hinge to the largest value no further than 1.5× the inter‐quartile range (IQR, that is, the distance between the first and third quartile). The notches extend to 1.58× the IQR divided by the square root of the number of participants, which gives roughly a 95% confidence interval for medians. The points correspond to individual values that lie beyond the whiskers. The asterisks denote means that are significantly different from each other (at *p* < 0.05).
**Figure S2: Box plots of the neural findings in the right TPJ (A) and lateral PFC (B, C) demonstrating significant differences in baseline current density (A/m** ^**2**^ **) between the three behavioral types.** This figure complements Figure 2 with additional statistical information. The box plot's lower and upper hinges correspond to the first and third quartiles (the 25th and 75th percentiles), the median is indicated by the thick horizontal line within the box plot. The whiskers extend from the hinge to the largest value no further than 1.5× the inter‐quartile range (IQR, that is, the distance between the first and third quartile). The notches extend to 1.58× the IQR divided by the square root of the number of participants, which gives roughly a 95% confidence interval for medians. The points correspond to individual values that lie beyond the whiskers. The asterisks denote means that are significantly different from each other (at *p* < .05)
