# Supplementary material for: Neural traits characterize unconditional cooperators, conditional cooperators, and noncooperators in group‐based cooperation
Source: Hum Brain Mapp. 2019 Jul 16;40(15):4508–17. doi: 10.1002/hbm.24717 (PMC6773361; doi:10.1002/hbm.24717)
Supplement: Supplementary file 1 — Table S1 ANOVAs with and without covariate time lag. Overview of the results of the ANOVAs testing the effect of behavioral types on neural baseline activation in the right TPJ (beta2), and the left LPFC (beta2 and beta3), calculated with and without including the time lag between the EEG and the behavioral data collection as a covariate. Table S2. ANOVAs with and without covariate gender. Overview of the results of the ANOVAs testing the effect of behavioral types on neural baseline activation in the right TPJ (beta2), and the left LPFC (beta2 and beta3), calculated with and without including gender as a covariate. Table S3: Cluster solutions. Overview of the allocation and the behavioral characteristics of the two cluster solutions. Figure S1: Box plots of the contribution (A), belief (B) and contribution‐minus‐belief score (C) of the three behavioral types. This figure complements Figure 1 with additional statistical information. The box plot's lower and upper hinges correspond to the first and third quartiles (the 25th and 75th percentiles), the median is indicated by the thick horizontal line within the box plot. The whiskers extend from the hinge to the largest value no further than 1.5× the inter‐quartile range (IQR, that is, the distance between the first and third quartile). The notches extend to 1.58× the IQR divided by the square root of the number of participants, which gives roughly a 95% confidence interval for medians. The points correspond to individual values that lie beyond the whiskers. The asterisks denote means that are significantly different from each other (at p < 0.05). Figure S2: Box plots of the neural findings in the right TPJ (A) and lateral PFC (B, C) demonstrating significant differences in baseline current density (A/m 2 ) between the three behavioral types. This figure complements Figure 2 with additional statistical information. The box plot's lower and upper hinges correspond to the first and third quartiles (the 25th and 75th perc [file HBM-40-4508-s001.docx]

Supplementary Materials for

Neural traits characterize unconditional cooperators, conditional cooperators, and non-cooperators in group-based cooperation

Short title: Neural traits of behavioral types in the Public Goods Game

Thomas Baumgartner^†^, Franziska M. Dahinden^†^, Lorena R.R. Gianotti, & Daria Knoch

Department of Social Psychology and Social Neuroscience, Institute of Psychology,

University of Bern, Switzerland

^†^The first two authors contributed equally to this work

**This file includes:**

- Table S1. ANOVAs with and without covariate time lag.
- Table S2. ANOVAs with and without covariate gender.
- Table S3. Cluster solutions.
- Fig. S1: Box plots of the contribution, belief and contribution-minus-belief score of the three behavioral types
- Fig. S2: Box plots of the neural findings in the right TPJ and lateral PFC demonstrating significant differences in baseline current density (A/m^2^) between the three behavioral types.

**Table S1. ANOVAs with and without covariate time lag.** Overview of the results of the ANOVAs testing the effect of behavioral types on neural baseline activation in the right TPJ (beta2), and the left LPFC (beta2 and beta3), calculated with and without including the time lag between the EEG and the behavioral data collection as a covariate.

| ***ANOVAs*** |  | ***with covariate***  ***‘time lag’***  *(df1=2; df2=128)* | ***without covariate***  ***‘time lag’***  *(df1=2; df2=129)* |
| --- | --- | --- | --- |
| Independent variable: *behavioral types*  Dependent variable:  *right TPJ, beta2* | *F* | 5.174 | 5.490 |
|  | *P* | 0.007 | 0.005 |
|  | explained variance | 7.5% | 7.8% |
|  | observed power | 82.0% | 84.3% |
| Independent variable: *behavioral types*  Dependent variable:  *left LPFC, beta2* | *F* | 5.886 | 6.066 |
|  | *P* | 0.004 | 0.003 |
|  | explained variance | 8.4% | 8.6% |
|  | observed power | 86.9% | 87.9% |
| Independent variable:  *Behavioral types*  Dependent variable:  *left LPFC, beta3* | *F* | 5.973 | 6.113 |
|  | *P* | 0.003 | 0.003 |
|  | explained variance | 8.5% | 8.7% |
|  | observed power | 87.4% | 88.2% |

**Table S2. ANOVAs with and without covariate gender.** Overview of the results of the ANOVAs testing the effect of behavioral types on neural baseline activation in the right TPJ (beta2), and the left LPFC (beta2 and beta3), calculated with and without including gender as a covariate.

| ***ANOVAs*** |  | ***with covariate ‘gender’***  *(df1=2; df2=129)* | ***without covariate ‘gender’***  *(df1=2, df2=130)* |
| --- | --- | --- | --- |
| Independent variable:  *behavioral types*  Dependent variable:  *right TPJ, beta2* | *F* | 5.490 | 5.120 |
|  | *P* | 0.005 | 0.007 |
|  | explained variance | 7.8% | 7.3% |
|  | observed power | 84.3% | 81.6% |
| Independent variable:  *behavioral types*  Dependent variable:  *left LPFC, beta2* | *F* | 6.066 | 6.026 |
|  | *P* | 0.003 | 0.003 |
|  | explained variance | 8.6% | 8.5% |
|  | observed power | 87.9% | 87.7% |
| Independent variable:  *behavioral types*  Dependent variable:  *left LPFC, beta3* | *F* | 6.113 | 5.983 |
|  | *P* | 0.003 | 0.003 |
|  | explained variance | 8.7% | 8.4% |
|  | observed power | 88.2% | 87.4% |

**Table S3: Cluster solutions.** Overview of the allocation and the behavioral characteristics of the two cluster solutions.

|  | ***Cluster analysis based on contribution*** | | | | ***Cluster analysis based on contribution and belief*** | | | |
| --- | --- | --- | --- | --- | --- | --- | --- | --- |
|  | *NC* | *CC* | *UC* | *NC* | | *CC* | *UC* |  |
| *N* | 32 | 75 | 26 | 34 | | 74 | 25 |  |
| *% of sample* | 24.1% | 56.4% | 19.5% | 25.6% | | 55.6% | 18.8% |  |
| *mean contribution* | 2.3 | 9.4 | 19.0 | 2.6 | | 9.5 | 19.2 |  |
| *mean belief* | 6.9 | 9.0 | 13.4 | 6.7 | | 9.1 | 13.5 |  |
| *mean contribution-minus-belief* | -4.6 | 0.4 | 5.6 | -4.1 | | 0.4 | 5.6 |  |

*NC = Non-cooperators / CC = Conditional cooperators / UC = Unconditional cooperators*

**
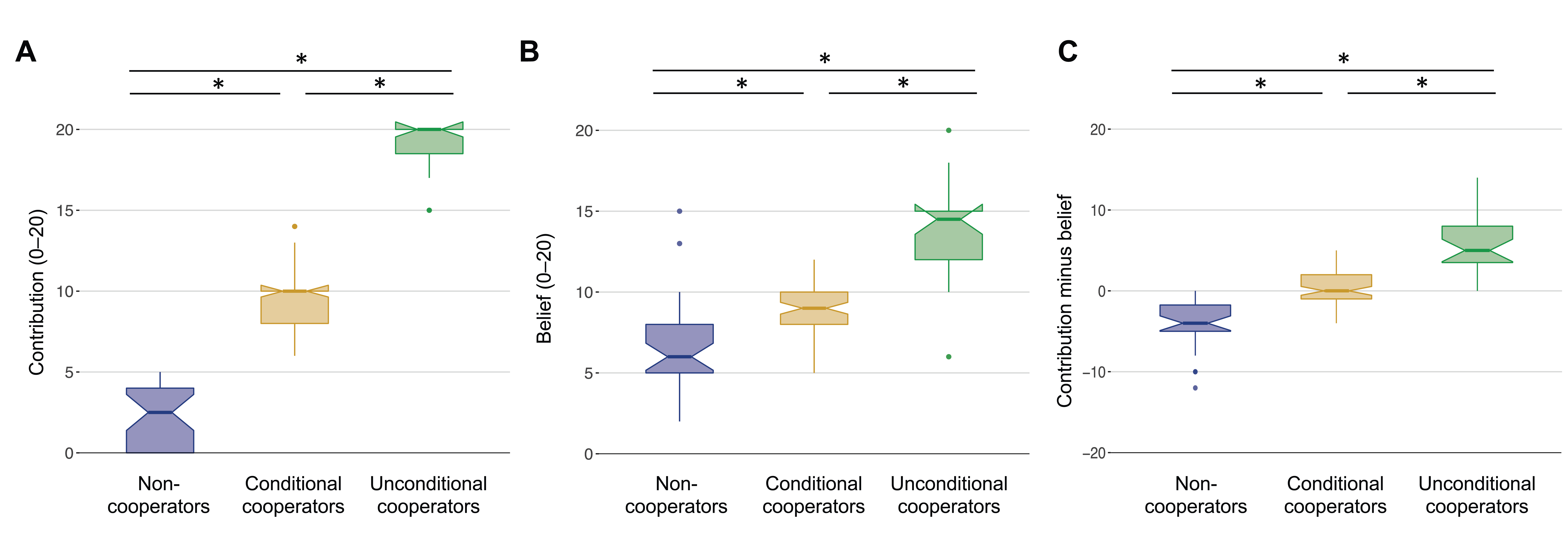
**

**Fig. S1: Box plots of the contribution (A), belief (B) and contribution-minus-belief score (C) of the three behavioral types.** This figure complements Fig.1 with additional statistical information. The box plot’s lower and upper hinges correspond to the first and third quartiles (the 25^th^ and 75^th^ percentiles), the median is indicated by the thick horizontal line within the box plot. The whiskers extend from the hinge to the largest value no further than 1.5× the inter-quartile range (IQR, i.e., the distance between the first and third quartile). The notches extend to 1.58× the IQR divided by the square root of the number of participants, which gives roughly a 95% confidence interval for medians. The points correspond to individual values that lie beyond the whiskers. The asterisks denote means that are significantly different from each other (at *P* < 0.05).

**
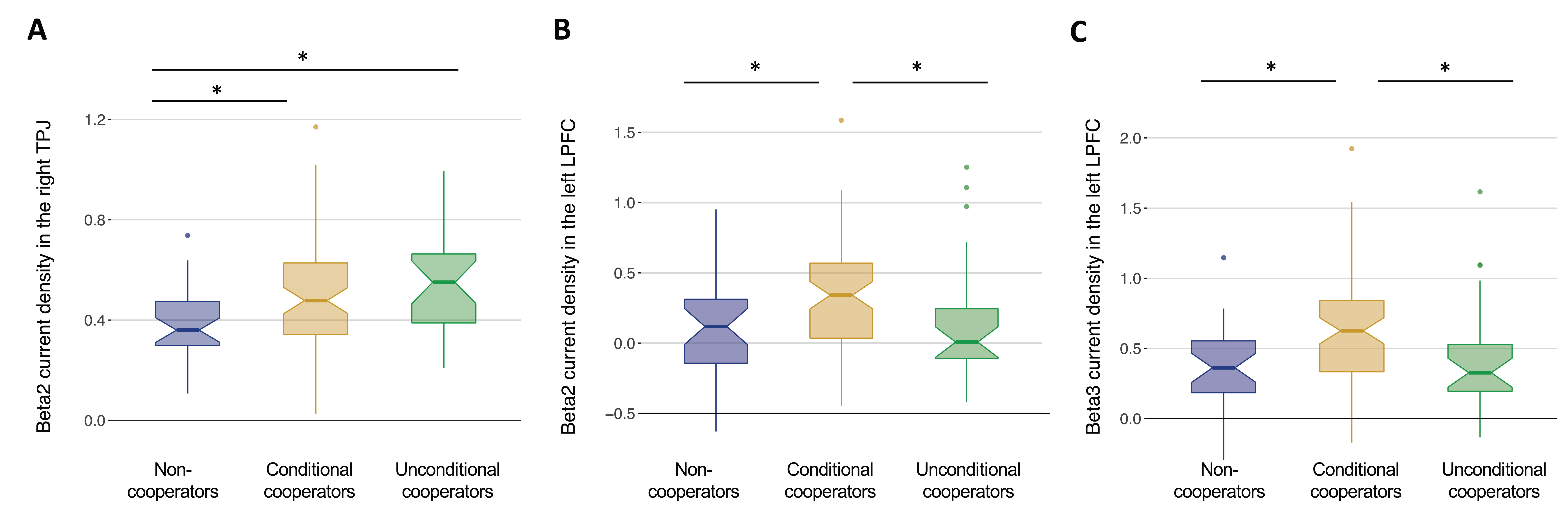
**

**Fig. S2: Box plots of the neural findings in the right TPJ (A) and lateral PFC (B, C) demonstrating significant differences in baseline current density (A/m^2^) between the three behavioral types.** This figure complements Fig.2 with additional statistical information. The box plot’s lower and upper hinges correspond to the first and third quartiles (the 25^th^ and 75^th^ percentiles), the median is indicated by the thick horizontal line within the box plot. The whiskers extend from the hinge to the largest value no further than 1.5× the inter-quartile range (IQR, i.e., the distance between the first and third quartile). The notches extend to 1.58× the IQR divided by the square root of the number of participants, which gives roughly a 95% confidence interval for medians. The points correspond to individual values that lie beyond the whiskers. The asterisks denote means that are significantly different from each other (at *P* < 0.05).
